# Cellulite orbitaire compliquant une pansinusite aigue: à propos d'un cas

**DOI:** 10.11604/pamj.2015.22.321.8215

**Published:** 2015-12-02

**Authors:** Issam Serghini, Amine El Moqqadem, Salah Bellasri, Jaouad Laayoune, Jalal Hamama, Mohamed Boughalem

**Affiliations:** 1 Pôle Anesthésie- Réanimation, Hôpital Militaire Avicenne, Faculté de Médecine et de Pharmacie, Université Cadi Ayyad, 40010 Marrakech, Maroc; 2 Service Anesthésie- Réanimation, 5^ème^ Hôpital Militaire Guelmim, Maroc; 3 Service de Radiologie, 5^ème^ Hôpital Militaire Guelmim, Maroc; 4 Service d'Ophtalmologie, 5^ème^ Hôpital Militaire Guelmim, Maroc; 5 Service de Chirurgie Maxillo-Fasciale et de Stomatologie, 5^ème^ Hôpital Militaire Guelmim, Maroc

**Keywords:** Pansinusite, orbitaire, antibiothérapie, chirurgie, pansinusitis, eye socket, antibiotherapy, surgery

## Abstract

Les cellulites orbitaires sont des affections peu fréquentes. Ces infections peuvent être secondaire à une infection oculaire, péri oculaire ou à une septicémie. L'origine sinusienne reste la plus fréquente. Le risque de graves complications mettant en jeu le pronostic fonctionnel et vital nécessite un diagnostic rapide et une prise en charge précoce. Nous rapportons le cas clinique d'une femme de 70 ans connue diabétique, qui a présenté une cellulite orbitaire secondaire à une pansinusite négligée. Le traitement était à la fois médical et chirurgical: antibiothérapie et drainage. L’évolution a été favorable au bout du septième jour. Nous essayons à travers ce cas clinique de souligner la gravité des infections orbitaires et leurs conséquences dramatiques en cas de retard de prise en charge.

## Introduction

La cellulite orbitaire secondaire à une pansinusiteest une affection ophtalmique rare. Elle représente 80% des complications des sinusites [1]. Elle est potentiellement grave mettant en jeu le pronostic fonctionnel voire vital [2]. Une prise en charge précoce et adaptée permet d'améliorer le pronostic redoutable de cette affection et d’éviter les séquelles visuelles. Nous rapportons l'observation d'une femme de 70 ans qui a présenté une cellulite orbitaire droite compliquant une pansinusite antérieure.

## Patient et observation

**Figure 1:**
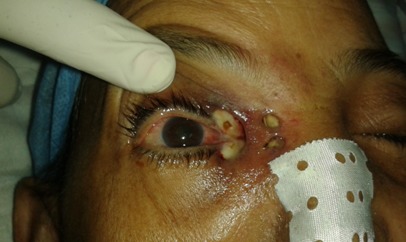
Tuméfaction orbitaire inflammatoire et douloureuse, une nécrose du cantus interne de l’œil

**Figure 2:**
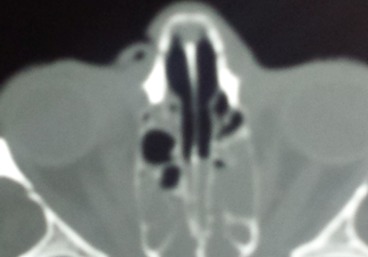
Perte de substance + bulle d'air au niveau du cantus interne de l’œil droit associé à un comblement du sinus sphénoïdal et des cellules ethmoïdales

**Figure 3:**
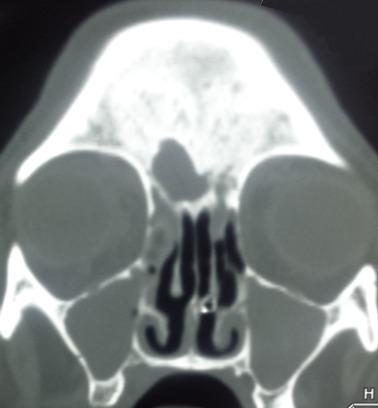
Pansinusite: comblement du sinus maxillaire et frontal

**Figure 4:**
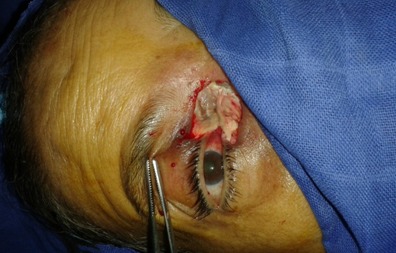
Necrosectomie au niveau du cantus interne de l'oe‘œil droit emportant le tendon palpébral médiane et une partie du muscle orbiculaire

Femme de 70 ans, immunodéprimée par un diabète de type I, a consulté pour un œdème palpébral droit et une rougeur périorbitaire évoluant dans un contexte fébrile depuis 06 jours. L'examen clinique trouvait une énorme tuméfaction orbitaire inflammatoire et douloureuse, une nécrose du cantus interne de l’œil, un ptosis, associée à une diplopie et trouble de l'oculomotricité etune fièvre à 3,5°C (Figure 1). L’évaluation de l'acuité visuelle était impossible vu l'intensité de la douleur. La patiente a été alors hospitalisée pour cellulite orbitaire. La Biologie a trouvé un syndrome inflammatoire: GB à 16 000 elt/mm^3^ - une CRP à 120 mg/l et une Glycémie à 3g/dl. La tomodensitométriecranio-orbitaire a montré une pansinusite avec épaississement des parties molles temporale gauche et du cantus interne de l'orbite droit avec respect des éléments intra-orbitaires (Figure 2,Figure 3). Sous anesthésie générale au bloc opératoire, la patiente a bénéficié d'une incision sous orbitaire droite, puis d'une necrosectomie au niveau du cantus interne de l’œil droit emportant le tendon palpébral médiane et une partie du muscle orbiculaire. La perte de substance consécutive est laissée sous cicatrisation dirigée (Figure 4). La patiente est mise sous tri antibiothérapie par voie intraveineuse pendant 5 jours puis relais par voie orale ceftriaxon 50mg/kg/j, gentamycine 3mg/kg, métronidazol 30mg/kg/j avec équilibration de son diabète. Après des soins et un examen ophtalmologique spécialisé quotidiens pendant sept jours, l’évolution était marquée par une nette amélioration de l'acuité visuelle et régression de la tuméfaction orbitaire.

## Discussion

Les cellulites orbitaires constituent les complications les plus fréquentes des sinusites aigues. Le point de départ est essentiellement ethmoïdal, plus rarement maxillaire et frontal. C'est une pathologie qui touche essentiellement les jeunes de moins de 15 ans [3] et l'adulte entre 60-70 ans avec une prépondérance masculine. Les germes les plus fréquemment observés chez l'adulte sont *Streptococcus pneumoniae et Staphylococcus aureus*. Chez l'enfant, on retrouve plutôt de Haemophilius influenzae, mais la vaccination a permis de réduire considérablement cette fréquence. L'immunodépression, essentiellement le diabéte, constitue un classique facteur favorisant la diffusion de l'infection [4]. Le tableau clinique associe une exophtalmie inflammatoire, un œdème palpébral, une diminution de la motilité oculaire.

L'imagerie est indispensable en cas de suspicion de cellulite orbitaire. Une tomodensitométrie orbitaire avec et sans injection de produit de contraste est l'examen clé du diagnostic positif et parfois étiologique [5]. Elle permet de déterminer la localisation exacte, la taille de la lésion orbitaire et l’état des sinus de la face.

La cellulite orbitaire vraie est une urgence. Il est important qu'elle soit reconnue précocement et traitée de façon énergique. C'est toujours une cause possible de cécité, voire de mortalité en cas de complication(s). Sur une petite série de 23 patients, Hodges et al. rapportent 52% de cécité et 4% de mortalité par thrombose du sinus caverneux [6]. Sur une série plus importante de 68 patients, Wane et al. rapportent 26% de cécité et là aussi, 4% de décès [7]. La cécité est secondaire à une neuropathie optique mécanique par élévation de la pression intra-orbitaire et/ou à une origine vasculaire par ischémie, une occlusion de l'artère centrale de la rétine, une thrombophlébite ou une origine inflammatoire (neurite infectieuse). Des occlusions vasculaires rétiniennes et/ou choroïdiennes, sources d'une baisse d'acuité visuelle, ont aussi été décrites. Enfin, une baisse d'acuité visuelle peut être liée à une kératite d'exposition due à l'exophtalmie ou plus exceptionnellement à des hémorragies et/ou exsudats rétiniens [8].

La prise en charge thérapeutique des complications orbitaires des sinusites aigues est une urgence. Elle repose toujours sur une antibiothérapie à large spectre. La chirurgie se justifie dans les formes collectées [9]. Les décongestionnants et les lavages nasaux par une solution isotonique ou hypertonique restent recommandés [10]. Les protocoles d'antibiothérapie proposés dans la littérature sont variés. La céphalosporine de troisième génération ou l'amoxicilline-acide clavulanique restent les molécules les plus préconisées. L'adjonction d'un antistaphylocoque majeure se justifie d'emblée ou en absence d'amélioration clinique en cas de suspicion d'un staphylocoque. Les imidazoles pourraient être associés particuliérement chez l'adulte [11].

Les nouvelles quinolones gagnent actuellement de plus en plus d'intérêt du faite de leur bonne diffusion tissulaire et leur rapidité d'action [12]. La durée de l'antibiothérapie intraveineuse est de 7 à 14 jours. Le relais per os est à envisager dés que l'apyrexie est durable, et aprés la disparition des signes inflammatoires locaux [13]. Cette antibiothérapie per os est d'autant plus longue que les signes cliniques ont mis plus longtemps à s'amender sous traitement parentéral [12]. La place de la corticothérapie dans le traitement des complications orbitaires d'origine sinusienne reste tou- jours débattue. Les corticoides en flash sont proposés en cas de suspicion d'atteinte du nerf optique [13]. Le drainage orbitaire, avec examen bactériologique, est indiqué en cas de collection orbitaire identifiée à la TDM ou devant l'aggravation clinique sous traitement médical bien adapté. Il est classiquement réalisé par voie externe via une orbitotomie médiale.

## Conclusion

La prise en charge de la cellulite orbitaire aiguë est une urgence. Son évolution est toujours grave en l'absence d'un traitement médical et chirurgical strict. Un scanner doit être immédiatement réalisé et une antibiothérapie parentérale débutée. En cas de résistance au traitement médical, une décompression par orbitotomie interne peut être nécessaire.
